# The effect of mannitol on oxidation‐reduction potential in patients undergoing deceased donor renal transplantation—A randomized controlled trial

**DOI:** 10.1111/aas.13713

**Published:** 2020-10-15

**Authors:** Christian Reiterer, Karin Hu, Samir Sljivic, Markus Falkner von Sonnenburg, Edith Fleischmann, Barbara Kabon

**Affiliations:** ^1^ Department of Anaesthesia Intensive Care Medicine and Pain Medicine Medical University of Vienna Vienna Austria; ^2^ Clinical Department of Nephrology and Dialysis Medical University of Vienna Vienna Austria

**Keywords:** deceased donor renal transplantation, mannitol, redox scavenging

## Abstract

**Background:**

Mannitol, an osmotic diuretic, is proposed to be an oxygen radical scavenger. Mannitol is often used in renal transplantation to attenuate oxidative stress and thus to protect renal graft function. We tested the hypothesis that mannitol reduces overall oxidative stress during deceased donor renal transplantation.

**Methods:**

We randomly assigned 34 patients undergoing deceased donor renal transplantation to receive a solution of mannitol or placebo shortly before graft reperfusion until the end of surgery. We evaluated oxidative stress by measuring the static oxidative‐reduction potential (sORP) and the capacity of the oxidative‐reduction potential (cORP). sORP and cORP were measured pre‐operatively, before and within 10 minutes after graft reperfusion, and post‐operatively.

**Results:**

Seventeen patients were enrolled in the mannitol group and 17 patients were enrolled in the placebo group. Mannitol had no significant effect on sORP (148.5 mV [136.2; 160.2]) as compared to placebo (143.6 mV [135.8; 163.2], *P = *.99). There was also no significant difference in cORP between the mannitol (0.22 µC [0.16; 0.36]) and the placebo group (0.22 µC [0.17; 0.38], *P* = .76).

**Conclusion:**

Mannitol showed no systemic redox scavenging effects during deceased donor renal transplantation. To evaluate the direct effect of mannitol on the renal graft further studies are needed.

*Trial registration:* ClinicalTrials.gov NCT02705573.

**Editorial Comment**

In the setting of ischemia‐reperfusion injury, reducing the levels of local reactive oxygen species could potentially be beneficial. This trial examined whether intravenous mannitol compared to saline would decrease the plasma sORP among kidney transplant recipients, but found no difference in the outcome.

## 1 INTRODUCTION

Renal transplantation is associated with ischemia/reperfusion (I/R) injury resulting in enhanced formation of reactive oxygen species (ROS), inflammation and activation of the innate immune system.^1^, ^2^, ^3^ ROS production is mainly released from three different systems: the nicotinamide adenine dinucleotide phosphate oxidase system, nitric oxide synthase system, and xanthine oxidase system.^3^, ^4^, ^5^, ^6^ Ischemia induced adenosine tri‐phosphate depletion leads to an increased hypoxanthine formation, which is converted to hydrogen peroxide (H_2_O_2_) and superoxide ($O_{2}^{‐}$).^4^, ^5^ Specifically, activated endothelial cells in ischemic tissue are major contributor to an overwhelming ROS release.^6^, ^7^, ^8^

Mannitol is an osmotic diuretic with proposed antioxidative capacity. Mannitol is commonly used in partial nephrectomy and renal transplantation to attenuate I/R injury.^9^, ^10^ However, there is only a small number of experimental animal studies evaluating the redox scavenging effects of mannitol.^11^, ^12^ Interestingly, a recent trial showed a significant attenuation of the post‐reperfusion syndrome in patients undergoing liver transplantation when mannitol was administered during liver reperfusion.^13^ Nevertheless, in patients undergoing deceased donor renal transplantation the radical scavenging effects of mannitol still remain unclear.

Therefore, we tested the primary hypothesis that the intraoperative administration of mannitol decreases overall static oxidative‐reduction potential (sORP) and increases overall capacity of the oxidative‐reduction potential (cORP) after surgery, as an indicator for a reduction in oxidative stress. As our secondary outcome we evaluated the percentage change of the oxidation‐reduction potentials after graft reperfusion between the mannitol and the placebo group.

## 2 METHODS

This study was approved by the Institutional Review Board (EK 2021/2014) of the Medical University of Vienna. Written informed consent was obtained from all patients participated in this trial. The trial was registered prior to patient enrollment at clinicaltrials.gov (NCT02705573, Principal Investigator: Samir Sljivic, Date of registration: 10 March 2016). The study was conducted according to the “Declaration of Helsinki” and followed the ICH GCP Guidelines.

We included patients with end‐stage renal disease between 18‐80 years undergoing deceased donor renal transplantation. Patients with known allergy to mannitol were excluded. All patients were hemodialyzed shortly before renal transplantation.

Donors after cardiac death were not included. Hypothermic machine perfusion of deceased donor kidneys was not performed.

### 2.1 Randomization

Patient allocated to the mannitol group received a 20% mannitol solution in a dose of 5 mL/kg bodyweight (BW) (concentration: 5 mL = 1 g).^14^ The placebo group received 0.9% NaCl solution in a dose of 5 mL/kg BW. The maximum dose of the study medication was restricted to 500 mL. In order to gain the maximum effect of mannitol we used a dosing regimen of 1 g/kg BW as previously described.^13^

A bolus of 100 mL of the study solution was administered shortly before graft reperfusion. The remaining study solution was infused till the end of surgery. A computer generated randomization sequence was created by an investigator, who was not involved in any trial procedures. The pharmacy, which provided our study medication, blinded and labeled the 34 bottles according to the randomization sequence. Every consecutive patient received one single bottle of 500 mL of the study solution in a consecutive ascending order. The patient, the attending anesthesiologist, and the research team were unaware of the group allocation. The randomization sequence was unblinded only for final data analysis at the end of the trial.

### 2.2 Protocol

Anesthesia was induced with 2‐3 μg/kg BW fentanyl and 2‐3 mg/kg BW propofol. Muscle relaxation was performed at the discretion of the attending anesthesiologist. Processed Electroencephalography (EEG)‐guided anesthesia was maintained with sevoflurane in 30% oxygen. Additional fentanyl was administered according to patient's requirements. We kept end‐tidal CO_2_ at near 35 mm Hg. Non‐invasive blood pressure was measured in 5‐minute intervals. Normothermia was maintained with forced‐air warming. According to clinical standards, all patients received a central venous line. Central venous blood gas samples were obtained hourly.

Fluid administration was esophageal Doppler guided (Cardio Q; Deltex Medical) according to a previous published algorithm.^15^ A balanced crystalloid solution (Elomel isoton; Fresenius Kabi) was used for intraoperative fluid replacement therapy. All patients received a baseline infusion rate of 2 mL/kg BW/h. We performed intraoperative esophagus Doppler guided goal‐directed fluid management (CardioQ; Deletex Medical). Fluid administration was based on the algorithm published by the Anesthesia Working Group of the ‘Enhanced Recovery after Surgery Society^15^ and was slightly modified. As compared to Feldheiser et al^15^ we used a lower intraoperative maintenance rate of 2 mL/kg BW and 250 mL for fluid bolus administration.

We placed the esophageal Doppler probe after induction of anesthesia. Once the characteristic Doppler signal was displayed, a fluid challenge of 250 mL was administered to assess stroke volume (SV) response. If the SV increased >10% (ie fluid responder), a further fluid bolus was administered. This was repeated as often as no further increase of more than 10% in SV was detected. In fluid non‐responders we treated coexisting hypotensive episodes, which were defined by a mean arterial pressure (MAP) < 70 mm Hg in normotensive and <80 mm Hg in hypertensive patients, with vasopressor titration at the discretion of the attending anesthesiologist.

Hemodynamic parameters were re‐evaluated at least every 15 minutes (or more frequently in case of significant hemodynamic changes, eg blood loss). When SV dropped more than 10%, we administered a further fluid bolus according to the above described algorithm.

Blood units were given as necessary. Transfusion trigger was a hemoglobin concentration of 7.0 mg/dL. However, if there was any clinical sign of organ hypoxemia (eg lactic acidosis) blood units were given earlier at the discretion of the attending anesthesiologist.

During the study period the additional use of further diuretics or drug treatment to reduce oxidative stress was not allowed.

### 2.3 sORP and cORP

We measured serum levels of oxidative stress using the RedoxSYS Diagnostic System (Aytu Biosience Inc) which were expressed as sORP and cORP as previously described.^16^

Thirty microliter of plasma were applied to the disposable sensors, which were inserted into the RedoxSYS Diagnostic System. Both parameters were provided after 4 minutes and recorded for analysis. All measurements were performed within 2 minutes of exposure to room air to avoid possible oxygen diffusion across the surface of the plasma influencing ORP results.

Plasma ORP is the measurement of the electron transfer from reductants (antioxidants) to oxidants under a constant negligible current (static ORP, sORP) and by increasing oxidative current (capacity ORP, cORP). Higher sORP (measured in millivolts, mV) values reflect the current redox balance and might correlate with illness, injury severity, and morbidity.^17^, ^18^, ^19^ cORP (measured in microcoulombs, µC) values provide an overview of the antioxidant reserves, which might correlate with the ability to respond to illness or injury.^17^, ^18^, ^19^

### 2.4 Measurements

Demographic data, comorbidities, renal replacement therapy, residual urinary output, long‐term medication, and pre‐operative laboratory values were recorded.

We received donor and organ specific information including age, gender, laboratory values, diuresis, and noradrenaline support from Eurotransplant. Cold and warm ischemia times were documented.

We measured duration of anesthesia and surgery, and vascular clamping times. Intraoperative fluid and hemodynamic parameters were recorded. Doses of anesthetics and vasoactive drugs as well as central venous blood gas analysis were also recorded.

Blood samples for sORP and cORP were drawn using serum‐lithium vacutainers. We took blood samples shortly before induction of anesthesia, before and after administration of the 100 mL bolus of the study medication and within 2 hours after surgery. Samples were centrifuged immediately at 1000 relative centrifugal force for 10 minutes at room temperature according to the user manual. We stored the serum at −80°C until further processing.

### 2.5 Statistical analysis

Statistical analysis was performed with IBM SPSS Statistics (Version 25). Mannitol and placebo study groups were compared for balance in patient characteristics, demographic data, pre‐operative laboratory values and kidney transplantation specific parameters. Normal distribution of the data was assessed, using a Kolmogorov‐Smirnov test. Normally distributed data were presented as mean ± standard deviation, not normally distributed data were given as median and 25th and 75th percentile. Chi‐square test was accomplished for comparing categorical variables. A *P*‐value < .05 was considered as statistically significant.

For the primary outcome was the effect of mannitol on overall sORP and cORP. Therefore, we performed a repeated measures linear mixed model between the groups for sORP and cORP after study solution administration.

As our secondary outcome we further analyzed the percentage change of sORP and cORP values from baseline using a repeated measures linear mixed model.

### 2.6 Sample size consideration

We estimated the number of patients required for this substudy based on a previous study evaluating the effect of partial liver resection on oxidative stress, which suggested that an increase of approximately 10% might be clinically relevant.^19^ The standard deviations of sORP ranged from 10 to 20 mV. Assuming a difference of 10‐15 mV between two treatment groups we calculated that 16 patients in each group have 90% power to detect a significant difference between the two groups at an alpha of 0.05.

## 3 RESULTS

**FIGURE 1:**
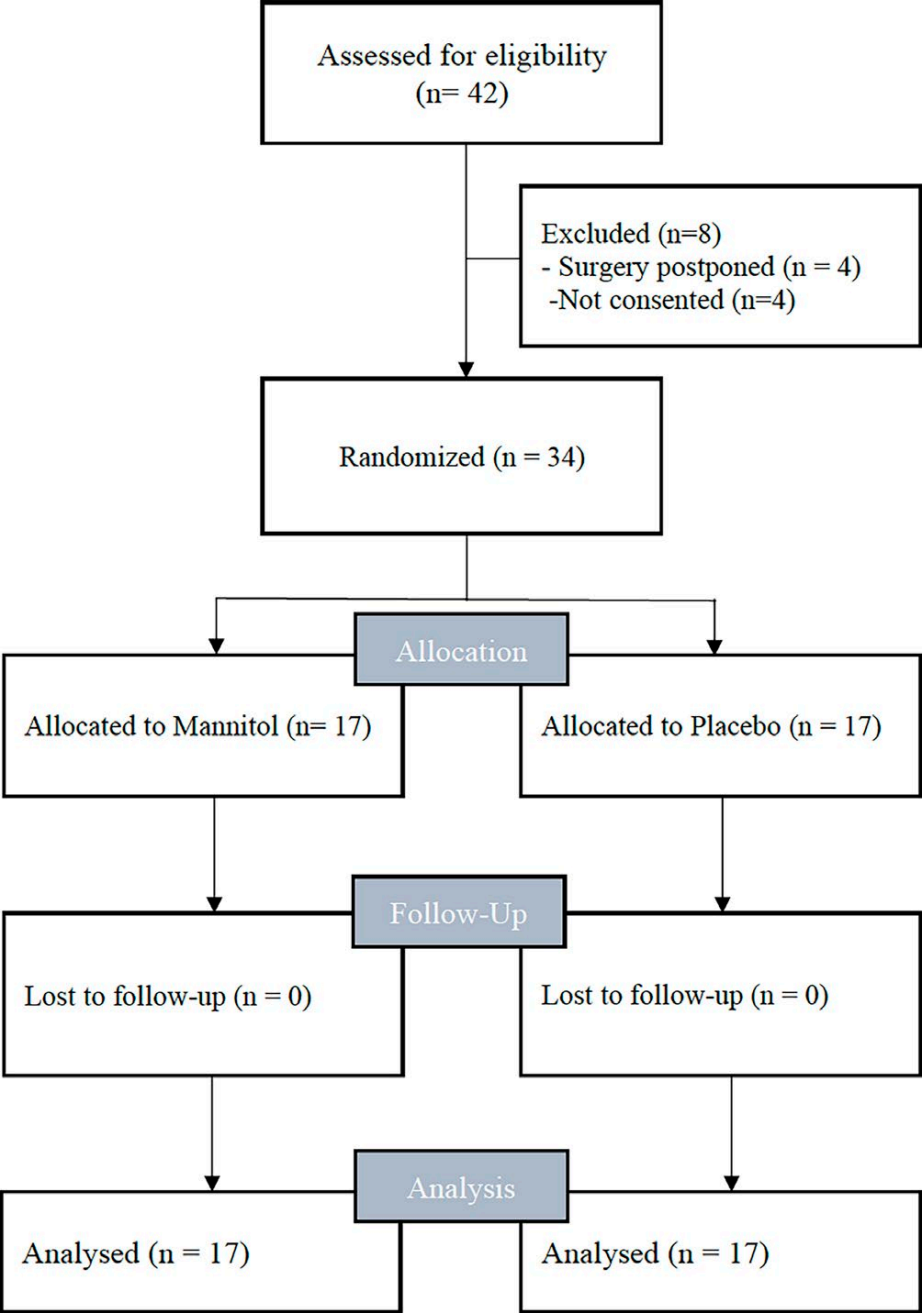
Consort 2010 patient flow chart

From January to July 2018 34 patients undergoing deceased donor renal transplantation were included at the Medical University of Vienna. Seventeen patients received the mannitol solution and 17 patients received the placebo (Figure 1).

Baseline characteristics, demographics, comorbidities, renal replacement therapy, urinary output, long‐term medication, and pre‐operative laboratory values did not differ significantly between both groups (Table 1). Donor specific data including age, gender, renal laboratory values, ischemia times, noradrenaline dosage and diuresis were similar between both groups (Table 1).

**TABLE 1:** Patient and donor baseline characteristics

	Mannitol (n = 17)	Placebo (n = 17)
Age, y	61 [57, 71]	54 [45, 71]
Height, cm	168 ± 10	170 ± 14
Weight, kg	76 ± 17	77 ± 17
Gender, no (%)		
Men	9 (53)	10 (59)
Women	8 (47)	7 (41)
Comorbidities, no (%)		
Hypertension	15 (88)	15 (88)
Pulmonary	2 (12)	6 (35)
Smoking history	5 (29)	3 (18)
Chronic intermit. Dialysis, no (%)	12 (71)	14 (82)
Peritoneal dialysis, no (%)	5 (29)	3 (18)
Residual urine output, mL/24 h	500 [0, 1000]	500 [0, 850]
Long‐term medication, no (%)		
Beta blocker	14 (82)	13 (76)
ACE inhibitors/AT1 blocker	8 (47)	10 (59)
Diuretics	5 (29)	6 (35)
Immunosuppressive therapy		
Tacrolimus, mg	4 [3, 4.5]	4 [3.5, 4.8]
Basiliximab, mg	20 [20, 20]	20 [20, 20]
MMF, mg	1000 [1000, 1000]	1000 [1000, 1000]
Pre‐operative laboratory values		
Hemoglobin, mg/dL	11.8 [10.7, 12.5]	11.3 [10.7, 12.5]
Creatinine, mg/dL	7.6 [5.6, 8,2]	8.2 [5.7, 9.8]
CRP, mg/dL	0.3 [0.1, 0.5]	0.3 [0, 0.7]
Donor characteristics		
Age, y	55 [51, 70]	60 [53, 71]
Gender, no (%)		
Men	9 (53)	11 (65)
Women	8 (47)	6 (35)
Laboratory parameters		
Creatinine, mg/dL	0.7 [0.6, 1.0]	0.9 [0.7, 1.0]
BUN, mg/dL	25 [14, 51]	19 [17, 46]
Diuresis, mL/24 h	2730 [1860, 4560]	2800 [2155, 4540]
Noradrenaline, mcg/kg/min	0.15 [0.07, 0.19]	0.16 [0.04, 0.22]
Duration of ischemia		
Cold, min	774 [501, 1030]	768 [543, 1148]
Warm, min	45 [35, 52]	44 [30, 58]

Note

Summary statistics of patient characteristics are presented as counts, percentages of patients, means ± SD, and median [25th percentile, 75th percentile].

Abbreviations: ACE, angiotensin converting enzyme; AT1, angiotensin 1; BUN, blood urea nitrogen; CRP, C‐reactive protein.

Intraoperative variables such as duration of anesthesia and surgery, clamp time, fluid balance and hemodynamic data and dosage of anesthetic‐ and vasoactive drugs were comparable. Averaged serum sodium values were slightly but significantly lower in patients receiving mannitol infusion (Table 2).

**TABLE 2:** Intraoperative variables

	Mannitol (n = 17)	Placebo (n = 17)	*P*‐value
Duration			
Anesthesia, h	3.5 (0.7)	3.7 (0.9)	0.56
Surgery, h	2.7 (0.6)	2.7 (0.8)	0.91
Arterial clamp, h	0.29 (0.10)	0.35 (0.13)	0.34
Venous clamp, h	0.37 (0.11)	0.35 (0.13)	0.92
Fluid and hemodynamics			
Total fluid, mL	1879 (541)	1824 ± 792	0.82
Bolus, no.	4 [3, 6]	5 [4, 6]	0.68
Blood loss, mL	303 [100, 675]	456 [0, 350]	0.39
MAP, TWA mm Hg	77 [75, 94]	79 [75, 81]	0.89
SV, mL	51 [41, 78]	64 [54, 72]	0.95
CO, L/min	3.1 [2.3, 4.5]	4.3 [3.2, 4.5]	0.80
CVP, mm Hg	13.1 ± 4.2	11.0 ± 4.4	0.41
Anesthesia variables			
Propofol, mg	200 [130, 200]	200 [100, 230]	0.89
Fentanyl, µg	650 [500, 750]	650 [450, 800]	0.76
TWA et Sevo, %	1.5 ± 0.3	1.5 ± 0.3	0.63
SpO_2_, %	98 [99, 100]	96 [96, 98]	0.06
Core, *T*°C	36.3 ± 0.5	36.4 ± 0.4	0.34
Phenylephrine			
No. of patients, (%)	9 (53)	13 (77)	0.28
Cumulative dose, mg	0.18 [0.00, 0.36]	0.13 [0.02, 0.20]	0.99
Noradrenaline			
No. of patients, (%)	5 (29)	5 (29)	1.00
Cumulative dose, mg	0.14 [0.00, 0.29]	0.11 [0.00, 0.20]	0.86
Central venous blood gas analysis			
pH	7.36 ± 0.1	7.38 ± 0.1	0.34
pCO_2,_ kPa	5.9 ± 1	6.1 ± 0.4	0.15
pO_2,_ kPa	6.5 [6, 7]	6.9 [6, 7]	0.47
Hb, g/dL	9.6 ± 1.4	9.6 ± 1.2	0.98
Na, mmol/L	136 ± 4	138 ± 2	0.03
K, mmol/L	4.7 ± 0.7	4.7 ± 0.4	0.88
Lactat, mmol/L	0.9 ± 0.2	0.9 ± 0.3	0.85

Note

Summary characteristics of intraoperative measurements presented as means (SD) or medians [25th percentile, 75th percentile]. All *P*‐values are for unpaired Student´s‐*t* tests or Mann‐Whitney‐*U* tests as appropriate.

Abbreviations: BE, base excess; CO, cardiac output; CVP, central venous pressure; FTc, corrected flow time; Hb, hemoglobin; K, potassium; MAP, mean arterial pressure; Na, sodium; pCO_2_, partial pressure of carbon dioxide; pO_2_, partial pressure of oxygen; SV, stroke volume; TWA, time weighted average.

**FIGURE 2:**
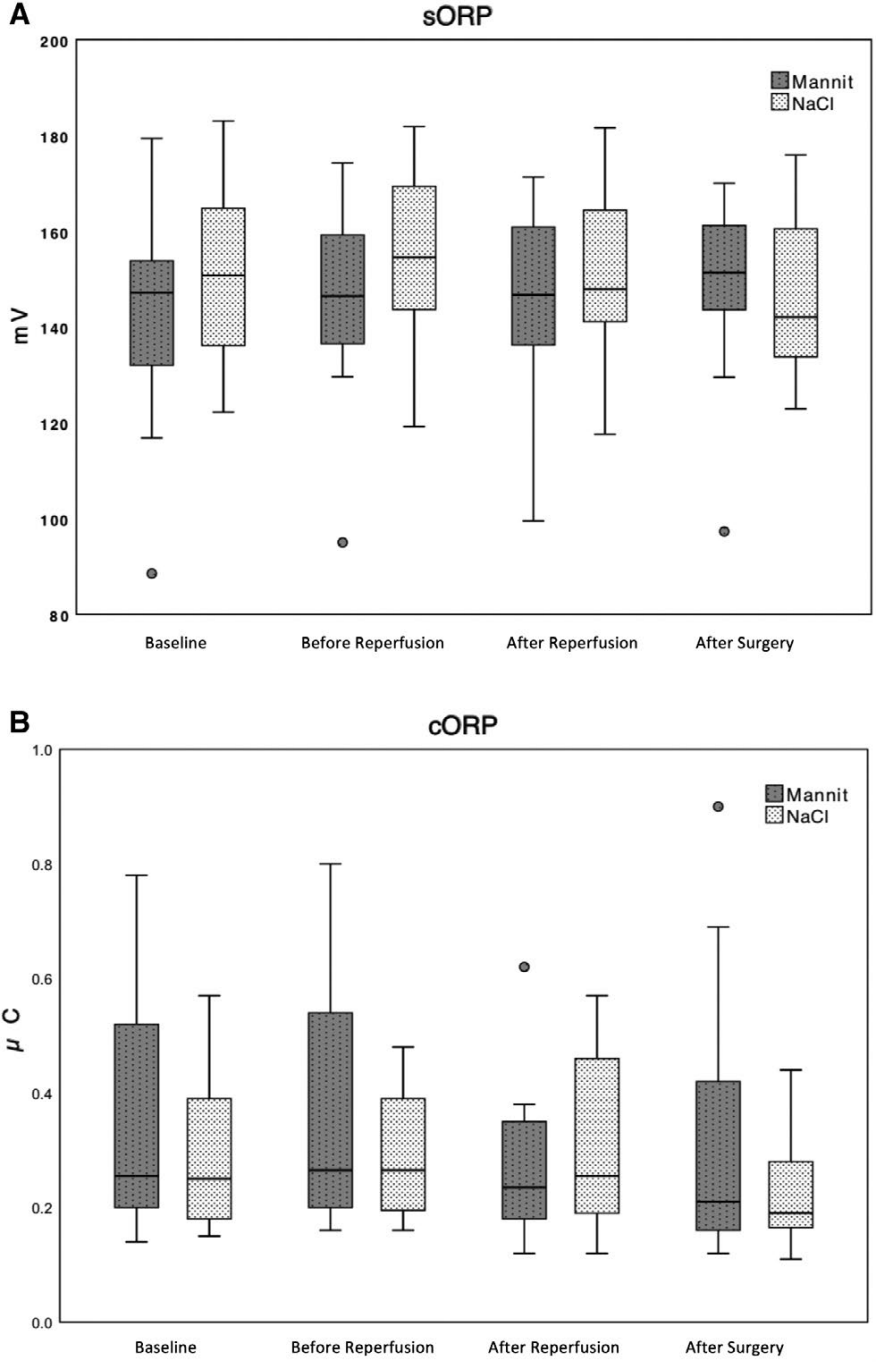
A, Box plots of sORP measurements of the mannitol group (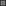) and the placebo group (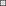) at baseline, before and after graft reperfusion and post‐operatively. Data are presented as median [25th percentile, 75th percentile], circles are presenting the extreme outliers. No differences were found between groups using a repeated measure mixed linear model (*P *= .99). B, Box plots of cORP measurements of the mannitol group (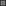) and the placebo group (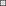) at baseline, before and after graft reperfusion, and post‐operatively. Data are presented as median [25th percentile, 75th percentile], circles are presenting the extreme outliers. No differences were found between groups using a repeated measure mixed linear model (*P *= .76). µC, microcoloumb; cORP, capacity of the oxidative‐reduction potential; mV, milli‐Volt; sORP, static oxidative‐reduction potential

Overall sORP after study drug administration did not differ significantly between the mannitol (155.3 mV [143.9; 163.9]) group and the placebo group (141.3 mV [132.4; 158.9], *P = *.99). There was also no significant difference in cORP after study drug administration between the mannitol (0.22 µC [0.16; 0.36]) and placebo group (0.22 µC [0.17; 0.38], *P* = .76) (Figure 2A,B).

Static oxidative‐reduction potential increased by 3.3% ± 8.9% in the mannitol group as compared to a decrease of 1.1% ± 7.8% in the placebo group (*P* = .09) (CI 95% −0.6 to 9.3). cORP decreased by 18.8% ± 44.8% in the mannitol group as compared to a decrease of −7.6% ± 43% in the placebo group (*P* = .29) (CI 95% −32.2 to 10.0).

## 4 DISCUSSION

Ischemia/reperfusion injury, mainly mediated by increased oxidative stress during organ transplantation, is associated with delayed renal graft function.^1^ We thus tested the capability of mannitol to scavenge free oxygen radicals and consequently to attenuate oxidative stress in patients undergoing renal transplantation.

In our study the administration of mannitol did not affect sORP and cORP values after renal graft reperfusion significantly. There were no differences in oxidation‐reduction potentials between baseline and post‐reperfusion values between the groups.

Static oxidative‐reduction potential and cORP give an overview of the systemic redox status in patients.^16^ It has been shown, that sOPR and cORP were markedly influenced in sepsis, increased systemic inflammation, infection, or during physiologic exercise.^16^, ^20^, ^21^ Furthermore, previous studies indicated that ORP values were associated with the occurrence of post‐operative complications. ^18^, ^19^ Moreover, oxidative stress plays an important role in morbidity and mortality, specifically in patients with cardiovascular comorbidities.^22^, ^23^

Therefore, future studies are needed to identify the predictive value of the oxidation‐reduction potential measurements; the integration into the clinical setting could be helpful for risk stratification and decision making.

Compared to a previous study investigating the effect of ORP on post‐operative complications in patients undergoing partial liver resection, our study population had higher baseline oxidative stress levels.^19^, ^24^, ^25^ A possible reason therefore might be, that we only included patients with end‐stage renal disease. The majority of our patients (seven of our patients received peritoneal dialysis) required intermitted renal replacement therapy. However, previous studies showed, that renal replacement therapy regardless of intermittend hemodialysis or peritoneal dialysis, are at risk for uremic acid accumulation. This strongly contributes to increased oxidative stress levels.^24^, ^25^, ^26^, ^27^

Several factors affect short‐ and long‐term oxidative stress levels after renal transplantations.^27^, ^28^ Immunosuppressive therapy, for example, was associated with increased ROS formation after renal transplantation.^27^ In contrast, normalization of renal function reduces long‐term ROS formation after renal transplantation.^28^, ^29^, ^30^ All of our patients received the first immunosuppressive therapy shortly before renal transplantation. As we only determined the short‐term effects of mannitol on the immediate perioperative ORP values, it seems unlikely that our measurements were affected by immunosuppressive therapy.

A recent study showed that mannitol significantly attenuated post‐reperfusion syndrome during liver transplantation.^13^ However, no biomarkers were measured to determine the oxidative stress level and these results should therefore be interpreted with some caution. The described oxidative scavenging effects were only based on differences in hemodynamic parameters between patients receiving mannitol and those receiving placebo. It is possible that the observed increase in cardiac ouput and SV might merely be mediated by the volume expanding effect of mannitol itself as rather by ROS scavenging effects. ^31^, ^32^

Previous studies indicated a significant impact of intraoperative fluid management on post‐operative renal graft function.^33^, ^34^ Thus, it is a strength of our study that we individualized intraoperative fluid‐ and vasopressor management according to patients’ requirements by using a previous published algorithm.^35^, ^36^ Specifically, we used esophageal‐Doppler guided fluid management to timely detect and correct hypovolemia to maximize SV. A further modifiable confounding factor for post‐operative renal function is arterial blood pressure, which serves as a major surrogate parameter for organ perfusion. Specifically, a MAP below 80 mm Hg was associated with delayed graft function after deceased renal donor transplantation.^37^ Recent trials showed that a MAP below 60‐70 mm Hg increases the risk of post‐operative acute kidney injury in noncardiac surgery.^38^, ^39^ Therefore, we tightly controlled intraoperative blood pressure with intravenous vasopressors, whenever SVs did not respond to further Doppler‐guided fluid bolus administration.

This study has several limitations. The main focus of our trial was the effect of mannitol on the systemic oxidative stress during renal transplantation. We did not perform blood sampling from the renal vein during and after reperfusion; thus, we cannot draw any conclusions regarding the effect of mannitol on I/R injury on the renal graft tissue per se. Furthermore, we did not measure ORP during the subsequent post‐operative days. Therefore, the long‐term effects of mannitol still remain unclear.

The ability of ORP markers to predict long‐term post‐operative renal function was not evaluated in this trial, but it might be crucial to be tested in further studies. Furthermore, our study was not powered to detect differences in clinical relevant outcomes like delayed graft function and the need of post‐transplantation renal replacement therapy.

We also did not evaluate the effect of mannitol on ORP in healthy patients. Results from patients without end‐stage renal disease might have been helpful to differentiate between the net effect of mannitol and other potential factors for example perioperative stress such as inflammation, infection/sepsis, immunosuppressive therapy or kidney function. Thus, the generalizability of our results remains speculative.

In literature the dosage of the administered mannitol for kidney injury prevention varies widely.^40^ However, to maximize potential effects of mannitol our chosen dosage was at the upper reported limit for this specific indication.^40^ Moreover, a similar dosing regimen has been proven to be safe and effective in attenuating post‐reperfusion syndrome.^13^

In summary, we did not show a significant effect of mannitol on sORP and cORP in patients undergoing renal transplantation. Because of our small sample size further studies are needed to confirm our results. Therefore, it remains for further interest how our data correspond to post‐operative delayed graft function.
